# Autotransplantation of a Third Molar to Replace an Adjacent Unrestorable Tooth: A Case Report

**DOI:** 10.7759/cureus.48133

**Published:** 2023-11-01

**Authors:** Saeed Asgary

**Affiliations:** 1 Iranian Center for Endodontic Research, Research Institute of Dental Sciences, Shahid Beheshti University of Medical Sciences, Tehran, IRN

**Keywords:** clinical dentistry, endodontics, cem cement, calcium-enriched mixture, autogenous tooth transplantation

## Abstract

Autogenous tooth transplantation (ATT) is a cost-effective and practical solution for managing severely compromised teeth, provided a suitable donor tooth is available. In this case report, we present a unique and successful ATT procedure performed on a 21-year-old female patient. The patient had an unrestorable adjacent tooth, which was replaced by a fully developed third molar. The procedure involved retrograde root canal filling using a calcium-enriched mixture cement, which took an extraoral time of eight minutes. The second molar was atraumatically extracted, and the mature third molar was immediately transplanted. A one-year clinical examination revealed a symptom-free patient with the transplanted tooth in proper occlusion, fully functional, and without any marginal periodontal issues. Radiographic assessments during follow-up appointments demonstrated bone regeneration, a healthy periodontal ligament, and an absence of external root resorption. This case report highlights the potential of mature third molar ATT combined with retrograde root canal filling as a promising approach to replacing lost permanent molar teeth, ultimately restoring both aesthetics and functionality.

## Introduction

Autogenous tooth transplantation (ATT) is a dental procedure that has seen increasing interest in recent years due to advances in protocols and a better understanding of the physiological impact of the periodontal ligament (PDL) during healing [1]. This method offers an alternative to single-tooth implantation and is particularly valuable for addressing tooth loss in young patients with developing alveolar bone, where traditional osseointegrated implants may not be ideal.

Successful ATT can lead to various benefits, including improved esthetics, arch form, dentofacial development, enhanced mastication, speech, and arch integrity [2]. The transplantation of third molars is crucial for preserving natural spaces, minimizing root resorption, maintaining alveolar bone volume, and shaping the morphology of the alveolar ridge through proprioceptive stimulation. However, success depends on careful case selection and a deep understanding of the biological principles involved [3]. Commonly, impacted maxillary canines and developing third molars are chosen for ATT. Third molars, when available, can serve as viable replacements for unrestorable or missing molars, presenting an attractive solution in dental treatments.

This case study presents a unique instance of using a mature upper third molar to replace an adjacent unrestorable second molar, with a comprehensive one-year evaluation to demonstrate the success and longevity of this innovative approach.

## Case presentation

A 21-year-old female patient presented with a complaint of an unrestorable crown on her upper left second molar. Her medical history was unremarkable, and a clinical examination indicated good oral hygiene. However, the second molar had a severely damaged crown, and radiographic assessment revealed a large carious lesion, making it beyond repair. The patient was informed of the compromised condition of the tooth, and extraction was recommended.

**Figure 1:**
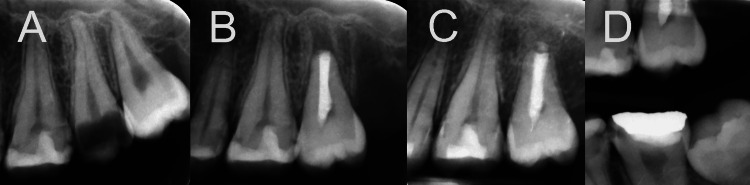
Radiographic progression of the treated case (A) Initial periapical radiograph shows the unrestorable second molar and the healthy third molar. (B) Radiographic image immediately after transplantation captures the immediate post-transplantation state. (C) One year after transplantation illustrates significant bone and periodontal ligament regeneration, with no evidence of root resorption. (D) Bite-wing radiograph one year after transplantation shows the establishment of a good occlusal interrelationship with the adjacent and opposing teeth.

Further examination revealed that the adjacent single-rooted third molar was in excellent health, fully developed, fully erupted, and well-positioned within the dental arch, making it an ideal candidate for ATT (Figure 1A). The patient was provided with detailed information about the treatment procedures, including associated benefits and risks. Informed consent was obtained, and the treatment was scheduled.

During the treatment session, oral disinfection was carried out using a 0.2% chlorhexidine gluconate solution. Local anesthesia was administered, and the procedure began with the atraumatic extraction of the third molar. After extraction, a root-end resection was performed, removing 2 mm from the apex. The root canal of the single canal was prepared using Gates Glidden burs #2-4 and irrigated with sterile normal saline solution. The canal was dried and subsequently filled and sealed with calcium-enriched mixture (CEM) cement. The unrestorable second molar was then atraumatically extracted. The wisdom tooth was repositioned in the recipient socket without interfering with opposing teeth. The transplanted tooth was stable and did not require fixation (Figure 1B). Remarkably, the entire procedure, from extraction to transplantation, was completed within a short eight-minute extraoral time.

The patient attended scheduled follow-up appointments for one day (post-operative check-up), one week, one month, and one year, including both clinical and radiographic assessments. The postoperative period remained uneventful. Radiographic and clinical evaluations at the one-year follow-up revealed the transplanted tooth in normal occlusion, with physiological mobility and effective masticatory function (Figure 1C and Figure 1D). Periodontal probing indicated no pockets or pathological signs, and the satisfied patient remained symptom-free. The PDL appeared intact, the periradicular area was healthy and normal, and there was no evidence of root resorption or periapical lesions.

## Discussion

The presented case report underscores the success and potential of ATT in replacing severely compromised teeth. In this instance, a fully developed third molar was endodontically treated in a retrograde manner and transplanted to replace a severely damaged adjacent second molar. The one-year follow-up revealed a symptom-free patient with functional and occlusally stable results, emphasizing the viability of mature third molar transplantation as an effective and promising approach for replacing permanent molar teeth and restoring both aesthetics and functionality.

ATT, with its roots dating back to ancient Egypt, continues to be a valuable and relevant approach in modern dentistry. Successful outcomes in ATT depend on careful case selection, adherence to biological principles, and the preservation of PDL cell vitality. The role of the PDL in the healing process and its physiological stimulation of alveolar bone volume are central to the success of ATT [3].

A notable feature of this case was the use of CEM cement for root canal filling, demonstrating its suitability for such procedures. CEM cement offers excellent sealing abilities, superior physical properties, and cost-effectiveness, making it a compelling option for endodontic treatments [4,5].

Efficient and precise surgical techniques, including atraumatic tooth extraction and immediate transplantation, were crucial aspects of this case. The remarkably short eight-minute extraoral time for the entire procedure minimizes the risk of complications and ankylosis, contributing to overall success.

The one-year follow-up assessments were encouraging, revealing normal occlusion, physiological mobility, and effective masticatory function. Moreover, there were no signs of periodontal issues or root resorption. Radiographic evaluation showed bone regeneration, a healthy PDL, and an absence of external root resorption, further confirming the success of the transplantation.

The patient's overall satisfaction and the longevity of the results indicate that mature third molar transplantation can be a promising approach for replacing unrestorable permanent molar teeth. However, it is important to note that successful ATT requires meticulous patient selection, appropriate donor teeth, and adherence to biological principles.

## Conclusions

This case report offers valuable insights into the successful ATT of a fully developed third molar to replace an unrestorable second molar. The efficient surgical procedure, utilization of CEM cement for retrograde root canal filling, and favorable one-year follow-up results underscore the potential of this approach for addressing severe dental issues. Further studies and long-term follow-ups are warranted to validate and refine this promising technique in dentistry.
